# Deficiency of Natural Plant-Derived Gut Metabolites Is a Potential Cause of Skeletal Deformities in Captive-Bred Green Peafowl (*Pavo muticus*)

**DOI:** 10.3390/ani16162495

**Published:** 2026-08-11

**Authors:** Deng Pan, Shengjia Sun, Yanqiu Chen, Wenji Tu, Bo Liu, Xiaoming Zhu, Ying Zhang, Xianghua Shu, Chunlian Song, Fang Li, Lin Lu

**Affiliations:** 1 College of Veterinary Medicine, Yunnan Agricultural University, Panlong District, Kunming 650201, China; pd@ynau.edu.cn (D.P.);; 2 Yunnan Forest Nature Center (Yunnan Wildlife Flora Rescue and Breeding Center), Panlong District, Kunming 650224, China; 3 Yubaiding Nature Reserve Management Bureau, Hongta District, Yuxi 653100, China

**Keywords:** green peafowl, ex situ conservation, dietary composition, gut metabolites, gut microbiome, bone homeostasis

## Abstract

Background: To recover endangered green peafowl (*Pavo muticus*) populations, China has implemented ex situ conservation programs centered on captive breeding. However, captive-bred individuals frequently suffer from skeletal deformities, creating a critical bottleneck that compromises future wild reintroductions. Results: This study demonstrates that, although green peafowl possess species-specific genes (*Igf2* and *Ndufs2*), they lack specialized allometric development of the cecum, suggesting that dietary disparities might play a more pivotal role in driving skeletal issues than innate intestinal anatomical factors. Specifically, wild peafowl naturally consume plants from the genus *Pistacia*, whereas captive birds receive a standardized diet dominated by *Glycine*. Reflecting these dietary disparities, captive-bred individuals exhibit significantly reduced gut levels of metabolites that regulate osteoclast activity, namely quercetin, taurine, urolithin A, and luteolin. Extensive evidence indicates that these specific metabolites directly modulate bone homeostasis during growth, meaning their reduction poses severe physiological risks. Furthermore, the abundance of these specific metabolites is directly modulated by gut bacterial communities, notably the genera *Escherichia* and *Staphylococcus*. We also found that dietary composition serves as a robust cue shaping gut microbiome assembly, acting independently of developmental stages. Conclusions: This study suggests that targeted plant supplementation holds promise in restoring the gut ecosystem and mitigating skeletal deformities, highlighting how diet-driven microbiome stewardship could advance the conservation and reintroduction of endangered species like the green peafowl.

## Simple Summary

Captive breeding is an indispensable strategy for protecting endangered species, but the health issues often experienced by captive-raised animals severely threaten their successful reintroduction into the wild. For the endangered green peafowl, one of the most serious hurdles is the frequent occurrence of skeletal deformities in captivity. In this study, we compared wild and captive birds to uncover how dietary differences affect their gut microbiome and bone health. We found that while wild green peafowl forage on a wide variety of natural plants, captive birds are fed a highly restricted diet. This lack of dietary diversity is associated with significant shifts in gut microbiota and reduced levels of plant-derived compounds potentially involved in bone development. Our findings suggest that these dietary shifts—rather than normal physical growth—are the primary factors disrupting the gut microbiome and impairing skeletal health. Ultimately, this research suggests that enriching captive diets with diverse, species-appropriate native plants may help mitigate skeletal deformities, improve animal welfare, and enhance the overall success of conservation and rewilding programs for endangered birds.

## 1. Introduction

While wildlife’s profound contributions to people are increasingly recognized, the accelerating loss of global biodiversity necessitates innovative conservation paradigms to protect vulnerable species [1,2]. Among these, the green peafowl (*Pavo muticus*), a species native to China, holds profound significance in conservation biology, Phasianidae phylogenetics, evolutionary characterization, physiology, ethology, and socio-cultural symbolism [3,4,5,6]. However, driven by rapid anthropogenic development [7], China’s wild green peafowl population has dwindled to an estimated 800–1000 individuals, necessitating captive breeding to bolster future reintroduction efforts. Biologically, animals such as the green peafowl are not merely autonomous entities but holobionts, representing a unified system of the host and its essential gut microbes [8]. Nevertheless, captive individuals frequently undergo functional degradation and gut dysbiosis during acclimation to artificial environments [9,10,11]. Because captive environments lack the dietary and environmental diversity of the wild, this dysbiosis essentially represents the degradation of the bird’s gut microecology [12,13]. Given that microbial communities profoundly influence host fitness, they should be recognized as a pivotal element in wildlife management and rescue practices [12].

In wildlife, specific geographic locations, genetics, and diet are all linked to the development of dysbiosis. Among these factors, dietary disturbances induced by captivity exert a more pronounced impact on the gut microbiota and its metabolites [13,14]. Generally, these impacts require two to three generations of community evolution to reach a steady state [15]. Notably, a healthy gut microbiota and its metabolites not only suppress the abundance of pathogens in animals but also participate in their physiological compensation [16]. Gut microbiota and their metabolites play essential roles in host immunity, energy homeostasis, and inter-organ crosstalk [17,18,19]. In avian species, skeletal deformities in captivity are classically attributed to physical constraints [20], insufficient B-wave ultraviolet exposure [21], and nutritional imbalances [22]. However, from an evolutionary perspective, an animal’s physical development heavily relies on chemical cues from its symbiotic microbes [23]. Emerging evidence highlights that alterations in gut microbial communities are prominent features in the onset and progression of skeletal diseases, involving dynamic interactions within the gut–bone axis or the gut–immune–bone axis [24]. Beyond pathological conditions, the gut microbiota maintains bone homeostasis through decomposition and metabolic activities [25]. Metabolites such as short-chain fatty acids (SCFAs), acting as crucial ecological signals linking the gut to the rest of the body, can regulate the species’ skeletal growth, mediated by factors such as insulin-like growth factors (IGFs) [26]. Furthermore, the gut microbiota indirectly regulates bone homeostasis by modulating immune cell differentiation balance, migration, and both innate and adaptive immune responses [27], while its food-derived biotransformation products (e.g., specific bacterial metabolites) can regulate osteoblast and osteoclast activity [28]. Therefore, under captive conditions, the attenuation or loss of these essential microbial signals in the green peafowl may directly compromise physiological functions, such as bone homeostasis.

Here, we investigated the microbial and metabolic alterations that may predispose captive green peafowl to a high risk of skeletal deformities. We collected fecal samples from wild and captive green peafowl during the same period, as well as from captive individuals at different developmental stages maintained under uniform dietary conditions. Our data indicate that the transition to captivity—encompassing profound dietary and environmental shifts—is strongly associated with alterations in the gut microbiota and the abundance of metabolites related to bone homeostasis, such as quercetin. Notably, the magnitude of structural shifts in the microbiome and metabolome driven by this wild-to-captive transition vastly supersedes the variations associated with ontogenetic development within the captive cohort itself. These findings suggest that changes in gut microbiota and metabolic profiles are associated with dietary shifts in captivity. Furthermore, these results support the view that successful wild bird conservation and reintroduction require careful consideration of microbiome stewardship [29].

## 2. Methods

### 2.1. Sample Collection of Green Peafowl

Fresh feces of wild green peafowl were collected within the jurisdiction of the Yubaiding Nature Reserve Management Bureau in Yuxi City, Yunnan Province, China. To ensure the independence and representativeness of the wild sample sources, 3 distinct individuals were simultaneously observed defecating from a distance by experienced reserve rangers using binoculars, and the fecal samples (*n* = 3) were collected immediately after the birds vacated the area. During the same period, fecal samples from captive-bred green peafowl, serving as controls, were collected at the Yunnan Forest Nature Center (Yunnan Wildlife Rescue and Breeding Center, *n* = 3).

To investigate differences in gut microbiota and metabolites among captive-bred green peafowl at different developmental stages under the same dietary composition, we divided the captive-bred individuals by age into a juvenile group (1–3 months old) and a subadult group (1–2 years old), which were housed separately in different isolation enclosures. To minimize stress interference caused by large-scale and frequent sampling, fecal collection for the juvenile group (*n* = 14) and the adult group (*n* = 14) was independently completed within two weeks. Immediately after collection, all fecal samples were snap-frozen in liquid nitrogen, transported on dry ice to the laboratory, and stored in a −80 °C ultra-low temperature freezer for subsequent use.

For cecal morphology studies, intestinal samples were collected from individuals ranging in age from hatchlings to subadults (*n* = 6). All samples were uniformly dissected to measure the absolute left cecal length and individual body mass, and the ratio of unilateral cecal length to body mass (mass-standardized relative cecal length) was then calculated. The allometric scaling of the green peafowl cecum was modeled using ordinary least squares regression and compared with a multi-species intestinal dataset provided by Duque-Correa et al. [30]. Prior to data fitting, both body mass and intestinal morphological data from all species were Log10-transformed to eliminate magnitude differences across datasets and to achieve linear fitting within the same coordinate system.

### 2.2. Species-Specific Gene Analysis of Green Peafowl

The genomic annotation information and gene identifiers for the green peafowl were collected from the published literature [31,32,33]. We extracted lists of positively selected genes and introgressed genes identified during the evolutionary divergence of blue and green peafowl from the aforementioned literature. To explore the biological functions of these genes, we utilized the Web-based GEne SeT AnaLysis Toolkit (WebGestalt v2024, https://www.webgestalt.org) to perform Kyoto Encyclopedia of Genes and Genomes (KEGG) pathway enrichment analysis [34], using the entire protein-coding gene set of *Gallus gallus* as the reference background. The enrichment analysis was conducted using a hypergeometric test, and the Benjamini–Hochberg method was applied for multiple testing correction, with a false discovery rate < 0.05 set as the significance threshold. Furthermore, to reveal the physical and functional associations among the proteins encoded by these genes, we constructed a protein–protein interaction (PPI) network utilizing the STRING database (v12.0, https://cn.string-db.org/) [35]. Due to the lack of a complete reference for the green peafowl in the STRING database, we used the closely related *G. gallus* as the reference species for homologous mapping and set the minimum required interaction score to medium confidence (>0.40). Subsequently, Cytoscape software (v3.9.1, http://www.cytoscape.org/) was used to visualize the constructed PPI network [36].

### 2.3. Dietary Analysis Based on Chloroplast Genome DNA Metabarcoding

Fresh wildlife fecal samples were subjected to ultra-low temperature preservation (−80 °C) immediately after collection, followed by total genomic DNA extraction using the FastDNA™ SPIN Kit for Soil (116564384, MP Biomedicals, CA, USA) specifically optimized for complex matrices. To exclude environmental and reagent contamination, negative blank controls were included concurrently set up during the extraction process. The concentration and integrity of the extracted products were assessed via fluorescence quantification and agarose gel electrophoresis. PCR amplification targeted the *rbcL-1* gene fragment in the chloroplast genome (primers: F: ATGTCACCACCAACAGAGACTAAAGC; R: CGTCCTTTGTAACGATCAAG). Three technical PCR replicates were performed for each sample to minimize amplification bias, and PCR negative controls were included. After magnetic bead purification of the amplified products, sequencing libraries were constructed, and paired-end 250 bp sequencing was executed on the Illumina NovaSeq 6000 platform (Illumina, San Diego, CA, USA).

Considering the potential amplification length polymorphisms of the *rbcL-1* target gene in complex environmental matrices, the raw demultiplexed sequences were imported into the QIIME 2 pipeline (v2025.4) and processed using a classic analytical strategy based on the Vsearch algorithm to maximize sequence retention rates [37]. After paired-end merging, quality trimming, and dereplication of the raw sequences, de novo chimera filtering was executed using the Vsearch plugin. Subsequently, the high-quality, non-chimeric sequences were clustered at a 97% similarity threshold to generate Operational Taxonomic Units (OTUs) and abundance data tables. For species composition analysis, the obtained OTU sequences were aligned against the NCBI GenBank database using the BLAST+ (v2.17.0) local alignment algorithm (identity ≥ 97%, E-value ≤ 1 × 10^−5^) to complete the taxonomic annotation of plant taxa, based on which the relative read abundance and occurrence frequency of each dietary source species were calculated.

To quantify diet structure, the OTU abundance table was rarefied, and α-Diversity indices (Chao1, Good’s coverage, and observed species) were calculated using QIIME 2 to evaluate the sequencing depth and intra-sample species richness. Differences in α-Diversity between groups were tested for significance using the Wilcoxon rank-sum test. For β-Diversity, structural variations in plant composition between samples were evaluated based on the Bray–Curtis distance matrix. Hierarchical clustering was visualized at the genus level utilizing the Unweighted Pair Group Method with Arithmetic Mean, and statistical differences in community structure between groups were tested via permutational multivariate analysis of variance (Adonis) [38]. Under default QIIME 2 parameters, random forest analysis was applied to discriminate samples from different groups, employing nested stratified k-fold cross-validation for automatic hyperparameter optimization and sample prediction (k = 5). The linear discriminant analysis effect size (LEfSe) method was employed with a logarithmic LDA score threshold of 3 to detect differentially abundant taxa among groups [39]. Simultaneously, the muma package (v1.4) in R (v4.3.3) was used to perform Orthogonal Partial Least Squares Discriminant Analysis as a supervised model to reveal variations in plant dietary composition across groups [40]. All *p*-values involving multiple comparisons mentioned above were corrected using the Benjamini–Hochberg method, with a significance threshold of *p* < 0.05.

### 2.4. LC-MS/MS and Gut Metabolome Analysis

A 20 mg fecal sample was weighed into a 2 mL centrifuge tube, and 300 μL of pre-cooled methanol (containing 5 ppm 2-chlorophenylalanine) was added. After grinding with a high-throughput tissue homogenizer (55 Hz, 60 s) and sonication (10 min), the samples were centrifuged at 12,000 rpm and 4 °C for 10 min; the supernatant was then passed through a 0.22 μm PTFE filter membrane, and the filtrate was collected into detection vials. A 20 μL aliquot of filtrate from each sample was pooled to create a quality control (QC) sample to evaluate instrument stability and data reliability. Liquid chromatography separation was performed utilizing an ACQUITY UPLC HSS T3 column (100 Å, 1.8 µm, 2.1 mm × 100 mm). The flow rate was set at 0.4 mL/min, the column temperature at 40 °C, the autosampler temperature at 8 °C, and the injection volume was 2 μL. In both positive and negative ion modes, mobile phase A consisted of 0.1% formic acid aqueous solution, and mobile phase B consisted of acetonitrile (containing 0.1% formic acid). The gradient elution program was as follows: 0–1 min, 5% B; 1–4.7 min, phase B linearly increased to 95%; 4.7–6 min, 95% B; 6–6.1 min, phase B decreased to 5%; 6.1–8.5 min, 5% B. Mass spectrometry analysis was conducted using an Orbitrap Exploris 120 mass spectrometer coupled with Xcalibur software V4.7 (both from Thermo Fisher Scientific, Waltham, MA, USA), performing data-dependent acquisition in positive and negative ion modes, respectively. A HESI ion source was utilized, with spray voltages of 3.5 kV and −3.0 kV in positive and negative modes, respectively. The sheath gas was set to 40 arb, auxiliary gas to 10 arb, capillary temperature to 320 °C, and auxiliary gas temperature to 300 °C. The full MS scan resolution was 60,000, mass range was 70–1000 m/z, AGC target was set to Standard, and maximum injection time (Max IT) was 100 ms. Following each full scan, the top 4 most intense precursor ions were triggered for secondary fragmentation (HCD collision energy 30%), with a dynamic exclusion time of 4 s, MS2 resolution of 15,000, and Max IT set to Auto.

The acquired .raw format data were imported into MS-DIAL software (v4.9.221218) for operations including peak extraction, alignment, filtering, and metabolite annotation [41]. Metabolic feature peaks with a detection rate of less than 50% in the QC samples were discarded; for undetected peaks, MS-DIAL software was used for missing value imputation, and data normalization was performed using the internal standard method. Metabolite identification was based on the Personalbio Next-Generation Metabolomics Database, which encompasses a standard self-built library, mzCloud database (https://www.mzcloud.org/), LIPID MAPS (https://www.lipidmaps.org/), HMDB (https://hmdb.ca/), MoNA (https://mona.fiehnlab.ucdavis.edu/), NIST_2020_MSMS, and an AI-predicted MSMS spectral library. The primary database search parameters were as follows: MS1 tolerance for identification 0.01 Da, MS2 tolerance for identification 0.05 Da, Smoothing level 3, Minimum peak height 10,000, Minimum peak width 5, Mass slice width 0.05 Da, and Identification score cut off 70. During data analysis, the screening criteria for differential metabolites between the two data groups were set to VIP > 1 and *p* < 0.05 (Student’s *t*-test). Following Z-score standard transformation of the differential metabolite abundance data, total differential metabolite clustering analysis was performed based on the Euclidean distance algorithm utilizing the pheatmap package (v1.0.12), and metabolites were categorized into different clusters according to the similarity of their expression patterns to conduct differential metabolite trend analysis. For the aforementioned differential metabolites, KEGG enrichment analysis was performed using the clusterProfiler package (v4.6.0) by mapping the annotated compounds to a custom KEGG compound background database. To explore their physiological significance, differential metabolites closely related to bone homeostasis were specifically extracted from the KEGG enrichment results. Their abundances were then Log2-transformed to facilitate clear visualization within a single coordinate system.

### 2.5. 16S rRNA Sequencing and Microbiome Analysis

To analyze the gut microbiota of wild and captive-bred green peafowl, primers were utilized to amplify the V3-V4 region of the bacterial 16S rRNA gene, and the forward and reverse primers were F: 5′-ACTCCTACGGGAGGCAGCA-3′ and R: 5′-GGACTACHVGGGTWTCTAAT-3′, respectively. A sample-specific 7-bp barcode was incorporated into the primers for multiplex sequencing. The PCR components were prepared in a 25 μL reaction volume and included 5 μL of buffer (5×), 0.25 μL of Fast Pfu DNA polymerase (5 U/μL), 2 μL of dNTPs (2.5 mM), 1 μL of forward primer (10 μM), 1 μL of reverse primer (10 μM), 1 μL of DNA template, and 14.75 μL of ddH2O. The thermal cycling protocol consisted of an initial denaturation at 98 °C for 5 min, followed by 25 cycles, each comprising denaturation at 98 °C for 30 s, annealing at 53 °C for 30 s, and extension at 72 °C for 45 s, with a final extension at 72 °C for 5 min. The PCR amplification products were purified utilizing Vazyme VAHTS™ DNA Clean Beads (N411, Vazyme, Nanjing, China) and quantified using the Quant-iT PicoGreen dsDNA Assay Kit (P11496, Invitrogen, Carlsbad, CA, USA). Subsequently, paired-end 250 bp sequencing was performed using the Illumina NovaSeq 6000 platform and the NovaSeq 6000 SP kit (Illumina, San Diego, CA, USA). Following data acquisition, the 16S sequencing data were imported into the QIIME 2 pipeline, relying on the DADA2 plugin to execute primer trimming, sequence quality truncation, error denoising, paired-end merging, and chimeric sequence filtering; taxonomic annotation of the gut microbiota was accomplished by aligning the obtained ASV representative sequences with the Greengenes 2 database.

The LEfSe method (LDA threshold = 4) was adopted to identify biomarker species with significant differences in relative abundance between groups. Correlation analysis between the differential microbiota and gut differential metabolites was performed utilizing R. After data processing, a chord diagram was plotted using the circlize package (v0.4.16), with a Spearman correlation coefficient threshold of 0.6 and *p* < 0.05, to elucidate the correlations between differential microbiota and gut differential metabolites associated with the regulation of bone homeostasis. Simultaneously, a Mantel test was conducted with default parameters using the vegan package (v2.6-6.1) to evaluate the correlation between the Bray–Curtis distance matrix of the differential microbiota and the distance matrix of bone homeostasis-associated gut differential metabolites. To analyze the impacts of developmental stages and dietary variations on the gut bacterial communities of green peafowl, a neutral community model (NCM) was fitted to the high-throughput sequencing-derived ASV data using non-linear least squares, and a Normalized Stochasticity Ratio model (NST) was concurrently constructed based on standard effect sizes.

### 2.6. Statistical Analysis of Breeding Performance and Health Outcomes

Statistical analysis and data visualization concerning breeding performance and health outcomes were performed using GraphPad Prism software (v9.5; GraphPad, San Diego, CA, USA). Continuous physiological variables, specifically eggshell weight, eggshell thickness, and hatchling body mass, were first assessed for normality using the Shapiro–Wilk test. Normally distributed continuous data are presented as the mean ± standard error of the mean (SEM), and statistical differences between the healthy (normal) and deformed cohorts were evaluated using Welch’s *t*-test, which inherently accounts for potential unequal variances. For data failing the normality assumption, the non-parametric Mann–Whitney *U* test was employed. The incidence of skeletal deformities among hatchlings, juveniles, and subadults is presented descriptively as percentages. Furthermore, survival probabilities post-hatching were estimated using the Kaplan–Meier method, and survival curves were statistically compared between healthy and deformed individuals using the Log-Rank (Mantel–Cox) test. The sample size (*n*) for each metric, representing biologically independent eggs or individual birds, is detailed in the respective figure legends. A two-tailed *p*-value < 0.05 was considered statistically significant.

## 3. Results

### 3.1. Severe Skeletal Deformities in Captive-Bred Green Peafowl

**Figure 1:**
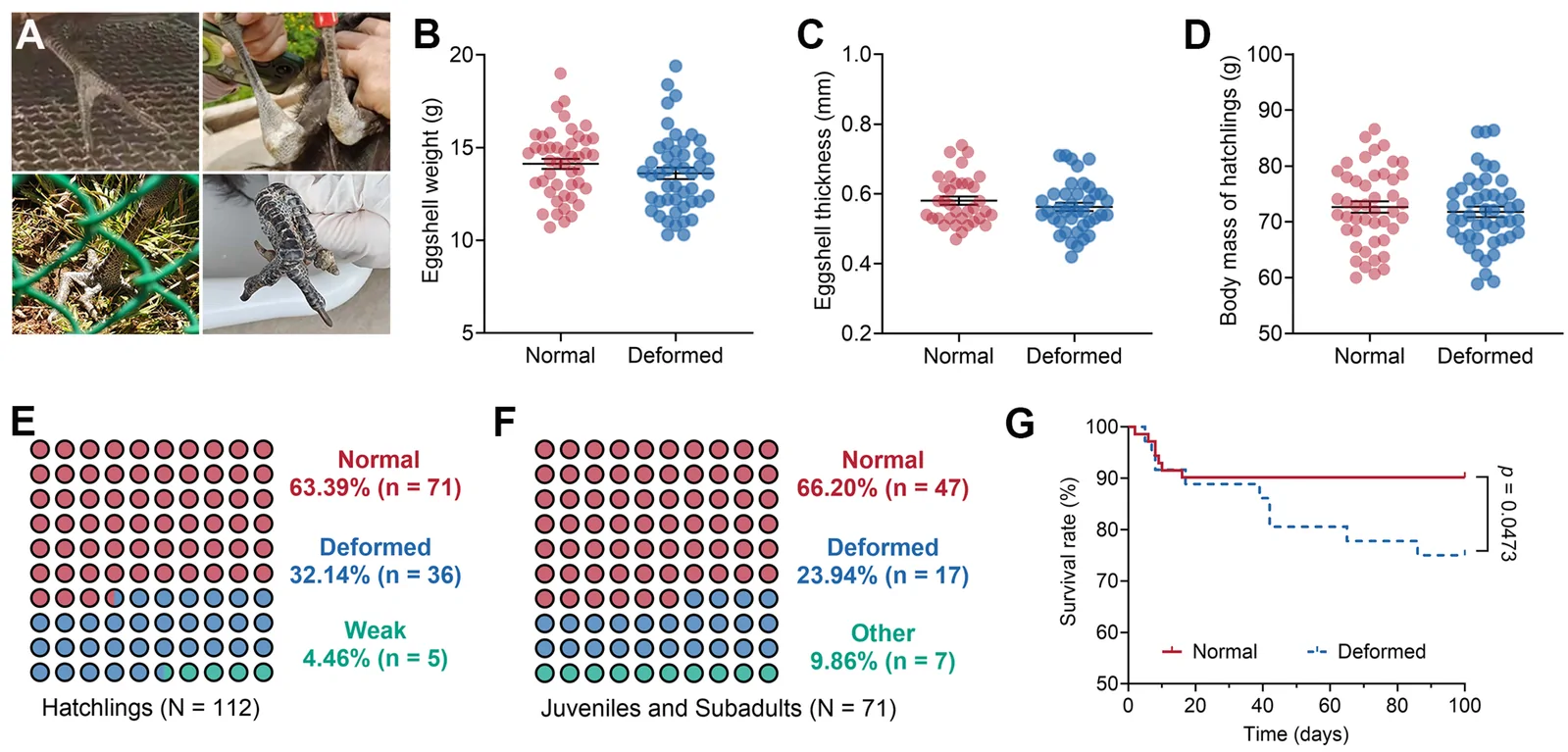
Skeletal deformities in captive-bred green peafowl. (**A**) Captive-bred green peafowl frequently exhibit varying degrees of skeletal deformities. (**B**–**D**) Eggshell weight (*n* = 46–47), eggshell thickness (*n* = 35–39), and hatchling body mass (*n* = 46–47) of captive-bred green peafowl during the 2024 and 2025 breeding seasons. (**E**,**F**) Proportion of individuals with skeletal deformities among hatchlings (**E**) as well as juveniles and subadults (**F**). (**G**) Survival curves of individuals with skeletal deformities (N = 107; excluding 5 weak hatchlings). Data were analyzed using Welch’s *t*-test (**B**–**D**) and the Log-Rank test (**G**).

In ex situ conservation settings, skeletal deformities are highly prevalent among captive-bred green peafowl, affecting both juveniles and subadults (Figure 1A). Prior to hatching, the fertilized egg functions as an independent biological unit, efficiently mobilizing calcium from the eggshell to support osteogenesis during embryonic development [42]. To investigate whether impaired calcium utilization contributes to this pathology, we analyzed eggshell metrics from captive-bred green peafowl collected between 2024 and 2025. Our findings revealed no significant differences in either eggshell weight or thickness between eggs from birds that subsequently developed deformities and those that remained healthy (*p* > 0.05; Figure 1B,C). Similarly, body mass did not differ significantly between the two groups at hatching (*p* > 0.05; Figure 1D). Despite these normal physiological parameters at hatching, the incidence of skeletal abnormalities remained high. Of the 112 captive-bred offspring, 36 individuals (32.14%) exhibited deformities upon hatching. During the subsequent brooding period, 17 of the remaining 71 initially healthy chicks (23.94%; excluding 5 weak hatchlings) also developed skeletal deformities (Figure 1E,F). Longitudinal monitoring from 2021 to 2025 revealed no significant difference in early-stage mortality between individuals with skeletal deformities and their unaffected counterparts. However, mortality rates increased significantly among affected individuals between one and three months post-hatching (*p* = 0.0473; Figure 1G). Collectively, these results suggest that skeletal deformities in green peafowl cannot be solely attributed to abnormal embryonic development or inefficient eggshell calcium mobilization. Instead, the condition appears closely linked to disrupted post-hatching bone homeostasis, which significantly compromises the overall fitness and survival of the birds.

### 3.2. Analysis of Green Peafowl Developmental Characteristics Based on Comparative Genomics

**Figure 2:**
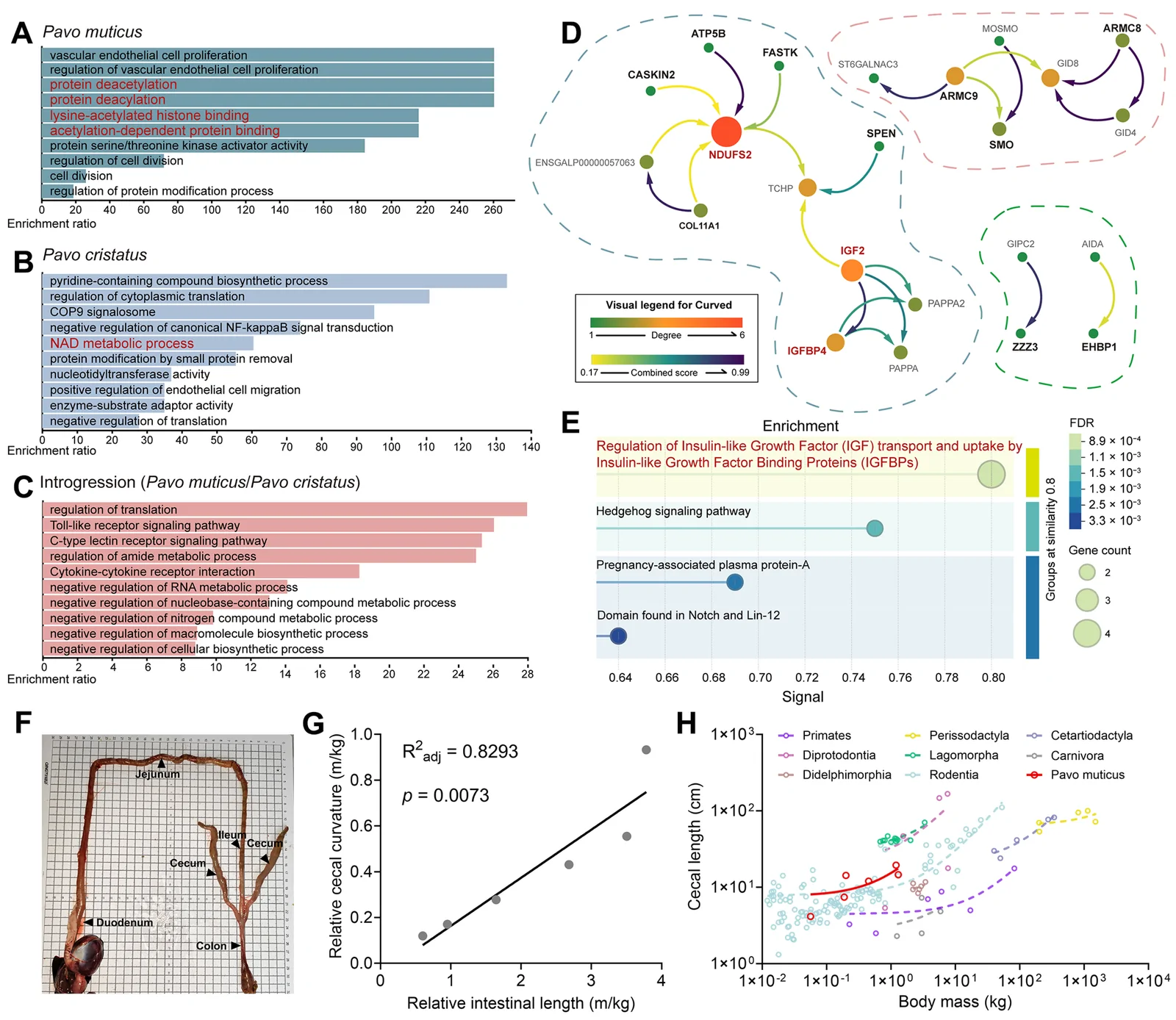
Enrichment analysis of positively selected genes during peafowl evolution and characteristics of cecal development. (**A**) GO and KEGG enrichment analyses of PSGs in the green peafowl (top 10). Pathways of interest highlighted in red. (**B**) GO and KEGG enrichment analyses of PSGs in the blue peafowl (top 10). Pathways of interest highlighted in red. (**C**) GO and KEGG enrichment analyses of introgressed genes between green and blue peafowl (top 10). (**D**) Protein–protein interaction PPI network mapping of PSGs in the green peafowl. PSGs are shown in bold. (**E**) Functional enrichment analysis of the PPI network. (**F**) Gross intestinal morphology of the green peafowl. (**G**) Ontogenetic morphological synchrony, demonstrated by regression analysis between mass-standardized relative cecal curvature (m/kg) and relative intestinal tract length (m/kg). (**H**) Comparison of cecal allometry between the green peafowl and other species, assessed via linear regression of Log10-transformed absolute cecal length versus body mass. Gene identifiers for the analyzed genes are derived from [31,32,33]. Gene enrichment analysis was conducted online using the WEB-based GEne SeT AnaLysis Toolkit (WebGestalt v2024, https://www.webgestalt.org). Cecal development data for certain animals were sourced from Duque-Correa et al. [30].

To investigate the underlying causes of the high incidence of skeletal deformities in captive-bred green peafowl, we analyzed potential species-specific genes using existing genomic evidence to gain insights from a molecular genetics perspective [31,32,33]. The positively selected genes (PSGs) identified in the green peafowl were found to be predominantly enriched in pathways related to protein deacetylation (GO:0006476, GO:0035601, GO:0070577, and GO:0140033) and the regulation of protein modification processes (GO:0031399; Figure 2A). In contrast, PSGs in the blue peafowl were associated with alterations in NAD metabolic processes (GO:0019674; Figure 2B). Although historical introgression events occurred between green and blue peafowls following their divergence, the functions of these introgressed genes were not enriched in the aforementioned pathways (Figure 2C). This indicates that these introgression events did not fundamentally alter the genetic divergence profiles between the two lineages at the genomic level.

Subsequently, the STRING database was utilized to construct a protein–protein interaction PPI network for the identified green peafowl PSGs. After filtering out genes that could not be accurately mapped in the avian database, insulin-like growth factor 2 (IGF2) emerged as a critical hub within the PPI network. It demonstrated a potential association with mitochondrial function (NDUFS2) but appeared to operate independently of the key deacetylation protein zinc finger ZZ-type containing 3 (Figure 2D). Functional enrichment analysis revealed that this network was primarily involved in the regulation of IGF transport and cellular uptake mediated by IGF-binding proteins (Figure 2E). These findings suggest that IGF2 represents a key species-specific gene driving the unique evolution of body size and skeletal development in the green peafowl [31]. This aligns with existing evidence demonstrating the regulatory role of IGF2 in vertebrate skeletal development [43].

Beyond determining body size, the biological activity of IGF2 also influences cecal dimensions [44]. In herbivorous birds, the products of cellulose digestion within the cecum are crucial for maintaining skeletal development [45]. This raises the question of whether the green peafowl has evolved an accelerated cecal growth rate to support its skeletal development. Anatomical examinations reveal that the cecum constitutes a substantial proportion of the total intestinal tract in the green peafowl (Figure 2F). By analyzing mass-standardized morphological metrics, we observed that cecal development is highly synchronized with overall intestinal elongation, exhibiting a robust phenotypic correlation across different age groups (R^2^_adj_ = 0.8635, *p* = 0.0073; Figure 2G). However, integrating evidence from Duque-Correa et al. [30] to assess true allometric scaling, we found that the allometric growth rate of the green peafowl’s cecum is not exceptional, falling between that of obligate herbivorous lagomorphs and certain omnivorous rodents (Figure 2H). This indicates that while IGF2 and the SCFAs derived from cecal cellulose digestion significantly impact avian skeletal development [46,47], the green peafowl’s capacity for cellulose digestion does not exhibit species-specific adaptations relative to other herbivores.

### 3.3. Dietary Composition Differs Between Wild and Captive-Bred Green Peafowl

**Figure 3:**
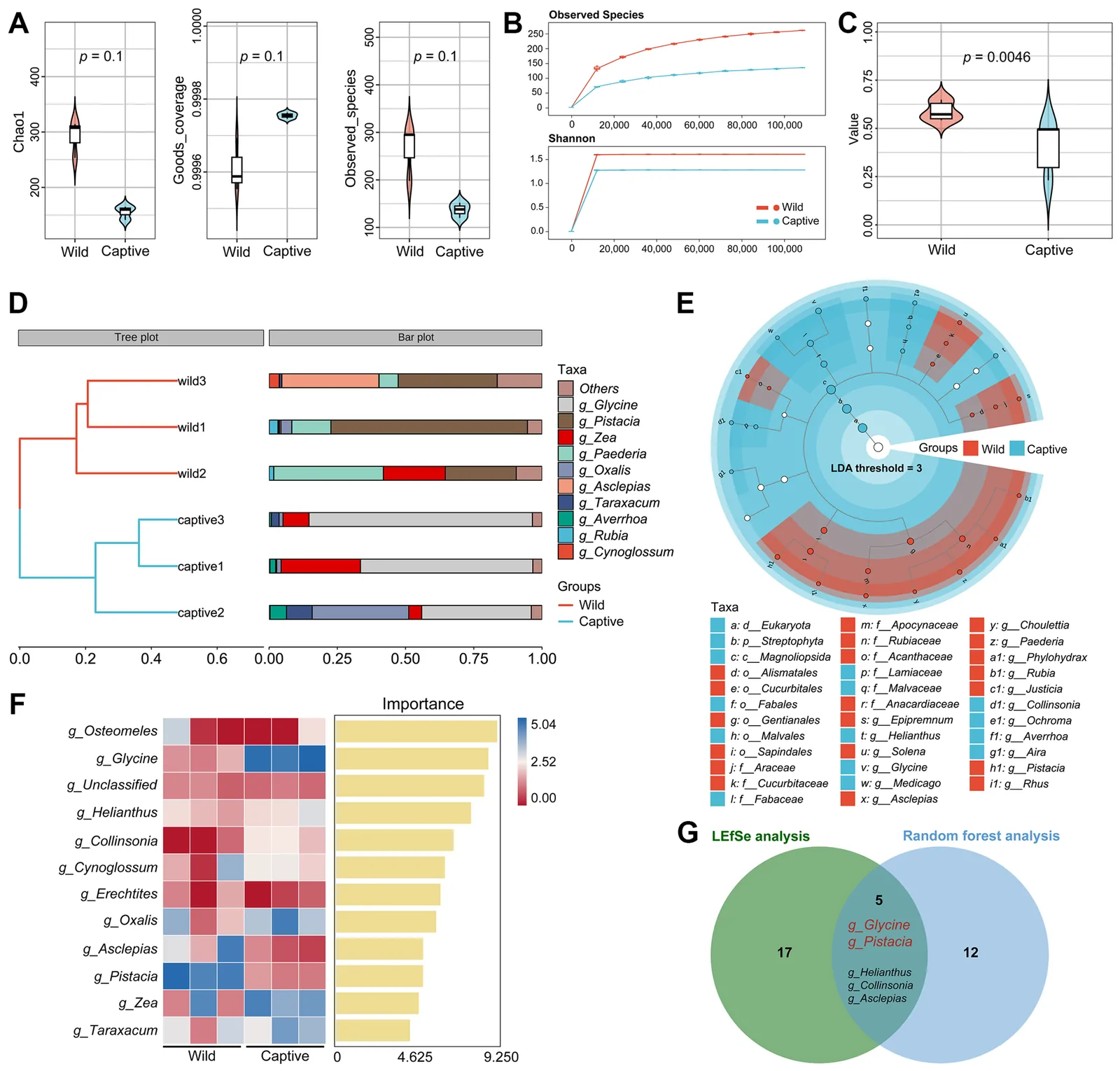
Dietary analysis of captive-bred and wild green peafowl based on fecal samples. (**A**) α-Diversity indices of plant species in fecal samples. (**B**) Rarefaction curves based on observed species and Shannon indices. (**C**,**D**) β-Diversity analysis of plant species in fecal samples, displaying intra-group distances (**C**) and hierarchical clustering (**D**). (**E**) Biomarker plant taxa characterizing dietary differences in green peafowl across different habitats. (**F**) Machine learning-based random forest analysis. (**G**) Overlapping key plant components identified by both LEfSe and random forest analyses. Red indicates shared and significantly differential plant taxa.

Next, we hypothesized that environmental factors might contribute to the skeletal development of the green peafowl. Evidence indicates that during ex situ conservation, alterations in environmental factors, such as management practices and resource availability, can affect bone homeostasis [48], with restricted food access being the most prevalent issue. Compared to their wild counterparts, although the richness of plant species consumed by captive-bred green peafowl fluctuated under restricted environmental conditions, this did not result in a significant difference in α-Diversity (Chao1: *p* = 0.1; Good’s coverage: *p* = 0.1; Observed species: *p* = 0.1; Figure 3A). Under the premise of confirming adequate sequencing depth (Figure 3B), the environment shaped stable differences in the structural composition of plant intake between captive-bred and wild individuals, significantly reducing the intra-group distance of β-Diversity among the captive-bred population (*p* = 0.0046; Figure 3C). Consequently, the dietary plant intake of captive-bred green peafowl exhibited greater homogenization.

Specifically, compared to wild green peafowl, captive-bred individuals consumed a higher proportion of plants from the leguminous genus *Glycine*, and fewer small trees from the anacardiaceous genus *Pistacia* (Figure 3D). Furthermore, LEfSe analysis based on DNA metabarcoding data of plant chloroplast genomes indicated that the top 10 most critical plant components consumed by captive-bred individuals originated from the genera *Glycine*, *Pueraria*, *Medicago*, *Rubia*, *Phylohydrax*, *Collinsonia*, *Quercus*, *Osteomeles*, *Choulettia*, and the family Apocynaceae. Conversely, the top 10 essential plant components for wild individuals were primarily derived from the genera *Paederia*, *Choulettia*, *Asclepias*, *Amalocalyx*, *Mangifera*, *Rhus*, *Pistacia*, an unclassified genus of Anacardiaceae (Unclassified_f_Anacardiaceae), *Fimbristylis*, and *Symplocos* (Figure 3E). Utilizing a machine-learning-based random forest model, we further validated the differential intake of these plant taxa between green peafowl in different habitats at the genus level (Figure 3F). In both the LEfSe and random forest analyses, the genus *Glycine* was consistently identified as a pivotal dietary species for captive-bred individuals, while the genus *Pistacia* was simultaneously confirmed as a key component in the diet of wild individuals (Figure 3G). These findings demonstrate that the diet of captive-bred individuals is predominantly composed of high-protein leguminous plants, whereas the diet of wild individuals is characterized by anacardiaceous plants, which contain higher levels of plant polyphenols.

### 3.4. Gut Metabolite Abundance Differs Between Wild and Captive-Bred Green Peafowl

**Figure 4:**
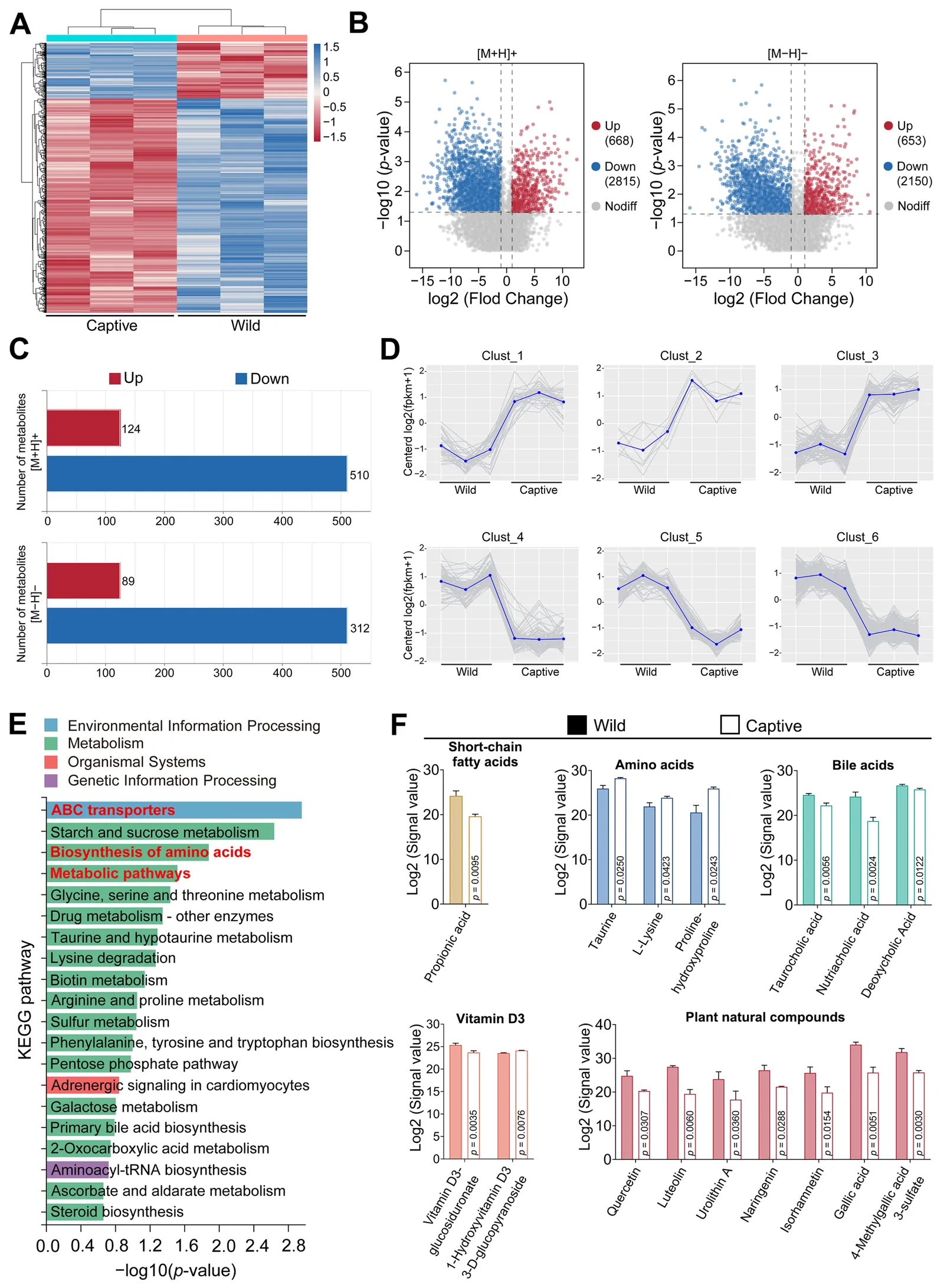
Untargeted metabolomic analysis of fecal samples from wild and captive-bred green peafowl. (**A**) Clustering analysis of total metabolites. (**B**) Volcano plot of differential metabolites (|Log_2_(Fold Change)| > 1, *p*-value < 0.05). (**C**) Number of successfully annotated differential metabolites. (**D**) Abundance dynamics and trend analysis of differential metabolites. (**E**) KEGG enrichment analysis of differential metabolites (top 20). (**F**) Differential metabolites associated with the regulation of bone homeostasis, corresponding to the KEGG terms shown in red.

During ex situ conservation, restricted dietary diversity can induce drastic shifts in the composition of animal gut metabolites [10]. Compared to their wild counterparts, the gut metabolic profiles of captive-bred individuals exhibit significant alterations (Figure 4A). Specifically, the abundances of 1321 metabolites were significantly increased, while 4965 were decreased (Figure 4B). Among these, 213 of the increased and 822 of the decreased metabolites were successfully annotated (Figure 4C). These differential metabolites were subsequently categorized into six distinct clusters based on their abundance dynamics (Figure 4D). They were primarily enriched in pathways associated with ABC transporters, carbohydrate metabolism, bile acid metabolism, amino acid biosynthesis, plant natural compounds, amino acid metabolism, and lipid metabolism (Figure 4E). Notably, differential metabolites closely associated with the regulation of bone homeostasis across three KEGG enrichment pathways are presented individually (highlighted in red; Figure 4E).

Compared to wild green peafowl, captive-bred individuals exhibited marked variations in the levels of propionic acid, taurocholic acid and its derivatives, as well as proline–hydroxyproline (an indicator of bone collagen degradation) (Figure 4F). Furthermore, although the abundance of certain vitamin D3 metabolites varied among green peafowl from different habitats, no significant alterations were detected in the levels of vitamin D3 itself or its primary active forms (Figure 4F). Notably, a substantial decrease in the levels of numerous plant-derived natural compounds and their metabolites was observed in captive-bred individuals (Figure 4F). These specific plant metabolic components have been previously demonstrated in vertebrates to influence osteoclast activation and reactive oxygen species (ROS) levels within bone tissue via the receptor activator of nuclear factor-κB ligand (RANKL) signaling pathway [49,50,51,52].

### 3.5. Integrated Analysis of Differential Gut Microbiota and Metabolites in Wild and Captive-Bred Green Peafowl

**Figure 5:**
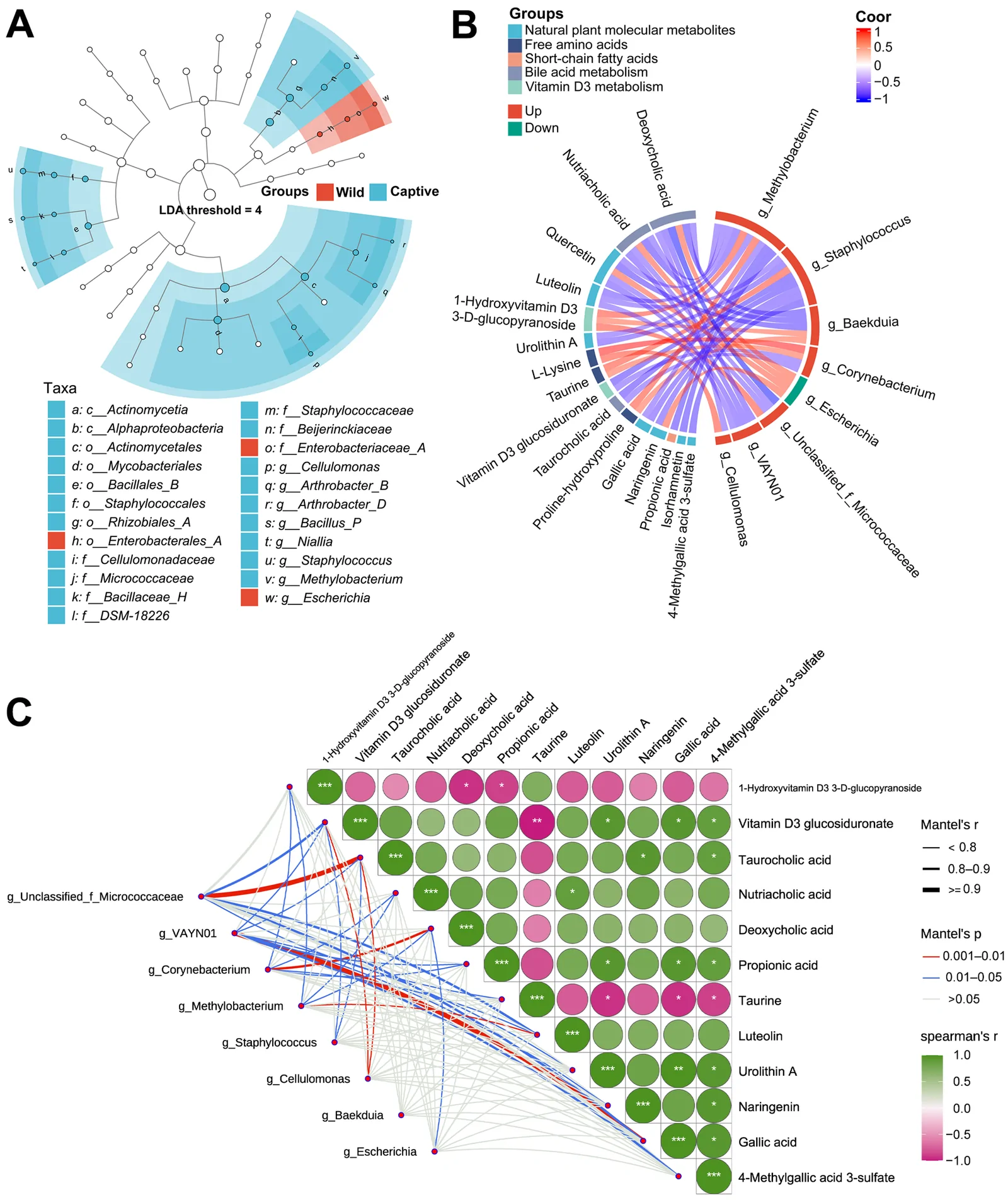
Integrated analysis of gut microbiota and metabolites in the green peafowl. (**A**) Biomarker differential bacterial taxa in the gut of wild and captive-bred green peafowl. (**B**) Integrated analysis of gut microbiota and major differential metabolites. (**C**) Consistency analysis of the associations between gut microbiota and differential metabolites. Statistical significance of the associations was indicated by asterisks: * indicates *p* < 0.05, ** indicates *p* < 0.01, and *** indicates *p* < 0.001.

Under altered dietary conditions, shifts in the gut metabolomic profiles of wild animals are frequently associated with gut microbiota dysbiosis [53]. Compared to wild individuals, the biomarker taxa enriched in captive-bred individuals included the family Cellulomonadaceae, as well as the genera *Arthrobacter_B*, *Arthrobacter_D*, *Bacillus_P*, *Niallia*, *Staphylococcus*, and *Methylobacterium*. In contrast, the genus *Escherichia* exhibited a significantly higher abundance in wild individuals (LDA = 5.2343, *p* = 0.0495; Figure 5A). Through an integrated analysis of microbiota and metabolites, we discovered that alterations in the abundances of the genera *Staphylococcus* and *Escherichia* were strongly correlated with fluctuations in the levels of gut metabolites, including taurocholic acid, taurine, quercetin, luteolin, urolithin A, and gallic acid (Figure 5B). These two genera, along with additional genera such as *Methylobacterium*, *Corynebacterium*, and *Cellulomonas*, collectively participate in the metabolism of the aforementioned plant-derived natural molecules (Figure 5C).

### 3.6. Dual Regulation of Peafowl Gut Microbiota and Metabolites by Growth and Diet

**Figure 6:**
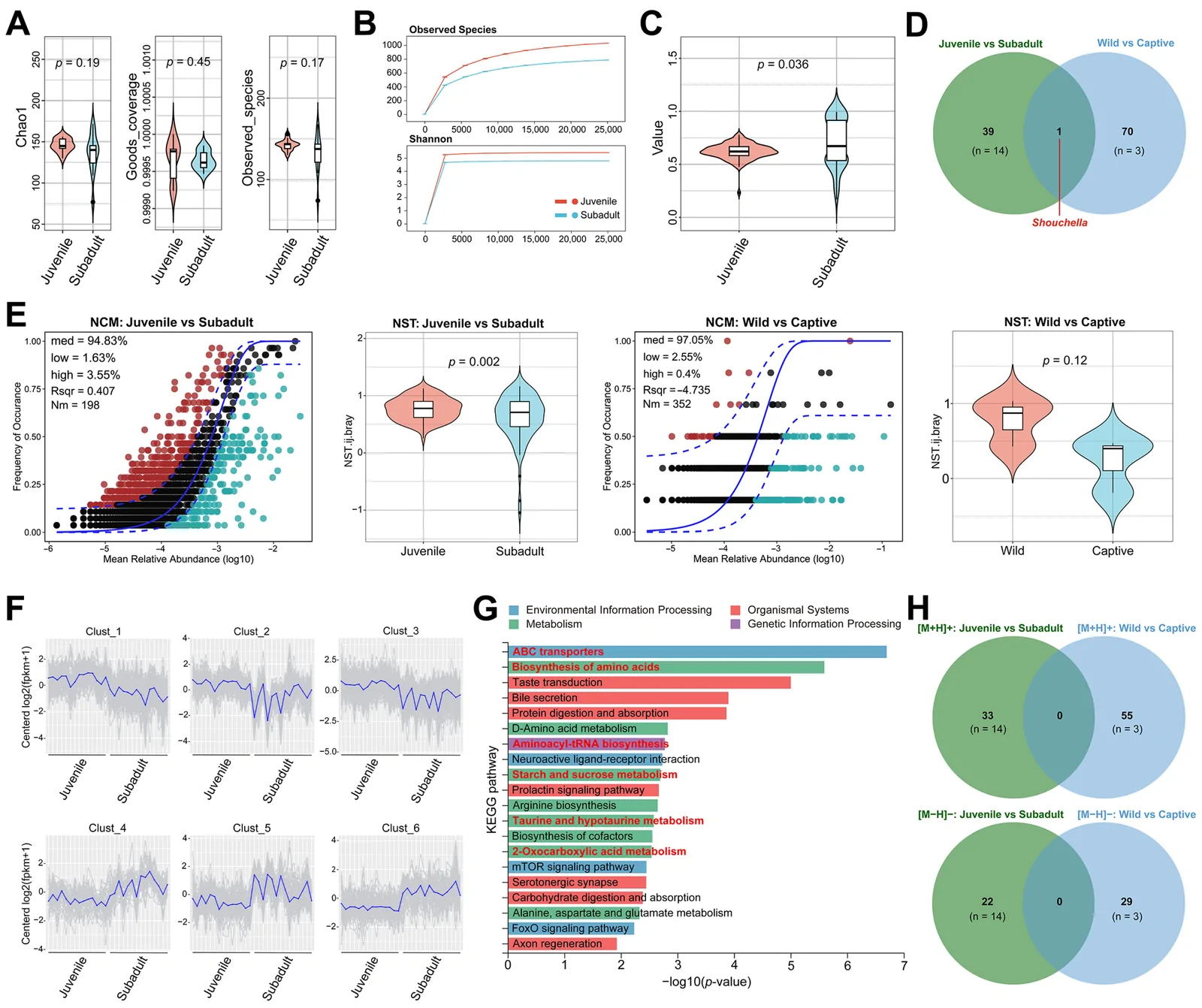
Independent regulation of the green peafowl gut microbiota and metabolites by growth development and diet. (**A**) α-Diversity analysis of gut microbiota in juvenile (1–3 months) and subadult (1–2 years) green peafowl (*n* = 14). (**B**) Rarefaction curves based on observed species and Shannon indices. (**C**) β-Diversity analysis of juvenile and adult green peafowl (*n* = 14). (**D**) Shared gut bacterial genera across different developmental stages (*n* = 14) and dietary compositions (*n* = 3). (**E**) Neutral community model (NCM) and normalized stochasticity ratio (NST) analyses of gut microbial community assembly in green peafowl across different developmental stages (*n* = 14) and dietary compositions (*n* = 3). Different colors indicate the occurrence frequency of taxa relative to the neutral prediction: red dots represent taxa occurring more frequently than predicted, teal dots represent taxa occurring less frequently than predicted, and black dots represent taxa fitting the neutral model. (**F**) Trend analysis of gut metabolites across different developmental stages (*n* = 14). (**G**) Functional prediction of gut metabolites at different developmental stages (*n* = 14). The KEGG terms shown in red represent shared terms across variations in developmental stages and dietary compositions. (**H**) Lack of overlap between gut metabolites identified across different developmental stages (*n* = 14) and dietary compositions (*n* = 3).

Gut microbiota and metabolic profiles are profoundly shaped by host development [54]. Here, we examined the gut microbiota and metabolites of green peafowl under captive conditions (representing a stable dietary environment) were examined. This allowed for a comparison with data obtained under different dietary conditions but equivalent climatic conditions, aiming to analyze whether growth development and diet independently regulate the assembly of the gut microbial community and metabolic profile in green peafowl. Overall, following the validation of sufficient sequencing depth, we found that the overall richness and evenness of the gut microbiota in green peafowl did not change across different developmental stages (Figure 6A,B). However, with respect to microbial community composition, significant differences were observed among captive-bred green peafowl individuals at different developmental stages (*p* = 0.036; Figure 6C). With the exception of the genus *Clostridium*, there was no overlap between the differential microorganisms identified across different developmental stages and those associated with different dietary states (Figure 6D). For this particular genus, there are currently no widespread reports indicating its involvement in bone homeostasis.

To explore the driving forces behind the alterations in microbial community composition, an NCM and an NST analysis were conducted to investigate whether microbiota shifts were dominated by a single variable. Across different growth stages, the assembly of the microbial community exhibited moderate stochasticity (NCM: R^2^ = 0.407). Conversely, among green peafowl under different dietary states, the community composition did not conform to the unified neutral theory (NCM: R^2^ = −4.735), indicating that diet, acting as a robust selective pressure (NST: *p* = 0.12), dominated microbiota assembly (Figure 6E).

Furthermore, the variance in gut metabolites among green peafowl individuals at different growth stages was not pronounced (Figure 6F). Although the differential metabolites identified across different growth stages and dietary states overlapped functionally (highlighted in red; Figure 6G), the specific metabolite entities were inconsistent (Figure 6H). Therefore, the gut microbiota and metabolites of the green peafowl are dually and independently driven by developmental stages and dietary factors. Diet, acting as a robust environmental filter, independently dominates the composition of the microbiota and the metabolic profiles in wild green peafowl.

## 4. Discussion

The issue of skeletal deformities in captive-bred green peafowl poses a severe threat to the conservation of this endangered species. These deformities not only elevate mortality rates during the critical 1 to 3 month post-hatching period for juveniles and subadults, but also compromise the overall success of reintroducing the species into its natural habitat. In commercial poultry and wild avian species, metabolic bone disease, perosis, and chondrodysplasia are typically attributed to absolute nutritional deficiencies or inappropriate environmental management [20,21,22]. However, in our study population, these traditional factors do not fully explain the observed phenotypic discrepancies. Mechanistically, there were no significant differences in eggshell quality (weight and thickness) or hatchling body mass between healthy and deformed individuals, indicating that the processes of maternal mineral mobilization and embryonic skeletal development remained relatively consistent.

Furthermore, while genetic bottlenecks and inbreeding depression are inherent risks for endangered populations under ex situ conservation, building upon previous comparative genomics evidences [31,32,33], we reveal an evolutionary signature in the green peafowl: an IGF2-centered regulatory network that drives its massive body size. Consistent with previous reports suggesting that IGF2 can elevate the basal activation level of osteoclasts [43]. Building upon this genetic foundation, our multi-omics results demonstrate that the primary alteration in the captive environment is not a simple mineral deficiency, but rather a severe dysregulation of the “gut-microbiota-bone” axis triggered by dietary shifts. The drastic reduction in specific plant polyphenols and amino acid metabolites—such as quercetin, taurine, and luteolin—deprives the organism of the essential chemical “brakes” required to inhibit excessive bone resorption [49,50,51,52]. Therefore, although traditional husbandry and management factors remain crucial, the disruption of host-symbiont metabolic signaling [55] constitutes a critical, previously unrecognized upstream trigger for skeletal deformities in captive green peafowl.

The regulatory mechanisms of bone homeostasis and the IGF2 system are conserved across vertebrates [43,56]. However, unlike in mammals, IGF2 and IGF2R do not exhibit genomic imprinting in avian species [57]. This subjects IGF2 to a more intense positive selection pressure during the evolution of the green peafowl, contributing to its massive body size [32]. Correspondingly, differences in growth performance driven by IGF2 have also been observed in chickens [58]. Regarding the maintenance of bone homeostasis, overexpression of IGF2 can influence signaling pathways such as NF-κB in osteoclasts via the IGF2 receptor, leading to hyperactivation of osteoclasts and subsequent bone loss [59]. As a binding ligand and negative regulator of IGF2, IGFBP4 has similarly undergone positive selection; it is predominantly expressed in low-strain regions such as bone joints and co-regulates osteoclast activity alongside factors like matrix metalloproteinase 9 [60]. Therefore, the positive selection of IGF2 in the green peafowl has not only shaped its immense body size but also contributed to an osteoclast activation level distinct from that of other Phasianidae species. From the perspective of functional adaptation, the degree of osteoclast activation is closely related to skeletal pneumaticity, which assists in reducing the organism’s energy metabolism [61]. Concurrently, given the bird’s massive size, this pneumaticity facilitates the capacity for short bursts of flight to evade predators and fulfills the requirements for nocturnal arboreal roosting. Nevertheless, bone resorption process in the green peafowl necessitates suppression by certain internal factors. Among the numerous evolved genes in the green peafowl, the proteins encoded by IGF2 and NDUFS2 (which participates in forming the hydrophilic arm of the NADH dehydrogenase complex) collectively serve as critical hubs in the PPI network. Compared to the blue peafowl, the secondary structure of the mitochondrial NADH dehydrogenase complex in the green peafowl underwent significant historical alterations. This may have led to drastically different population trajectories for the green and blue peafowl due to potential energy production deficiencies or mitochondrial abnormalities [62]. Furthermore, regarding bone resorption, the NAD^+^/NADH redox reaction has been proven crucial for osteoclast activity. Maternal supplementation with NAD precursors has been shown to effectively rescue fetal skeletal deformities at birth [63]. Consequently, to balance the multifaceted demands of body size development, flight capability, and bone homeostasis, osteoclast activity in the green peafowl must be moderately suppressed.

Diet-derived metabolites can modulate pathways associated with osteoclasts. It is well established that diet-induced shifts in the diversity and abundance of gut metabolites co-regulate organismal homeostasis alongside genetic factors [64]. Among these, gut microbial metabolites (such as short-chain fatty acids, bile acids and their derivatives, and amino acids) and extracellular vesicles, exert direct effects on bone remodeling, or indirectly influence osteoblast and osteoclast functions through immune-mediated pathways [65]. Regarding direct regulation, urolithin A and taurine primarily affect gene transcription by modulating ROS levels within osteoclasts. Specifically, urolithin A modulates osteoclastogenesis mainly by inducing mitophagy [66,67], whereas taurine functions primarily as a superoxide anion and free radical scavenger to maintain redox balance, thereby inhibiting transcriptional drive at the promoter level [51]. Although quercetin and luteolin are both flavonoids, their mechanisms of action differ. Quercetin directly suppresses activation of the NF-κB pathway to reduce osteoclastogenesis [68], while luteolin exerts a natural inhibitory effect on the interaction between the a3 and d2 subunits of V-ATPase, disrupting the osteoclast proton pump and impairing bone resorption [69]. In terms of indirect regulation, propionic acid not only downregulates osteoclast-related gene expression through glycolysis and oxidative phosphorylation under metabolic reprogramming [70], but also targets and inhibits the *Foxp3* gene via the GPR43-cAMP-PKA pathway and histone deacetylases deacetylation, thereby regulating T_reg_ cell populations [71]. Subsequently, IL-10 and TGF-β suppress NFATc1 in osteoclasts and block the NF-κB pathway, respectively, mitigating the degree of RANKL-induced osteoclast activation [72,73]. Therefore, wild green peafowl likely rely on a broad spectrum of plant-derived gut metabolites to concurrently inhibit NF-κB-regulated gene transcription in osteoclasts, suppress ROS elevation during osteoclast activation, and impede proton pump activation during bone resorption. This collective action effectively prevents IGF2 from overactivating osteoclasts via its receptor. However, in captive-bred green peafowl, this critical regulatory mechanism is significantly compromised.

Gut microbiota can impact avian bone homeostasis through the gut–bone axis and other gut-organ axes [74], representing a highly conserved yet ecologically vulnerable physiological feedback loop under captive conditions [24]. Fluctuations during different developmental stages and dietary availability can both induce shifts in the gut microbiota [75,76]. Notably, the driving effect of dietary availability on gut microbial community assembly in wild green peafowl is highly robust. This is likely due to the green peafowl’s strong dependence on water sources and plant richness [77,78]. Furthermore, our dietary analysis, we found that the primary differential plants consumed by wild green peafowl individuals were from the genera *Paederia* and *Pistacia*. A long-term specific diet can drive the population expansion of specific bacteria, and such traits are continually reinforced through inheritance [79]. Correspondingly, the abundance of taxa rich in β-glucosidase, such as the genus *Escherichia*, was found to be enriched in wild individuals [80]. This correlates with the higher content of glycoside derivatives in *Paederia* and *Pistacia* plants, such as quercitrin, quercetin-3-O-β-glucoside, and luteolin-7-O-glucoside (a luteolin precursor) [81,82]. Additionally, microbial communities rich in bile salt hydrolase, such as the genera *Staphylococcus* and *Bacillus*, also undergo changes to regulate the metabolic abundance of taurocholic acid and taurine [83], thereby participating in the modulation of osteoclast activity [84]. Although the gut microbiota and metabolites of wild animals are influenced by seasonality, the captive environment causes the gut microbiota and metabolites of captive-bred individuals to remain in a long-term stable state [85]. This suggests that dietary supplementation with *Pistacia* plants represents a promising strategy that warrants experimental evaluation in future conservation programs.

## 5. Conclusions

Our multi-omics evidence suggests that dietary plant richness is associated with gut microbiota composition and the presence of specific plant-derived metabolites, which may contribute to maintaining skeletal homeostasis in wild peafowl. However, these findings remain correlative at this stage. Therefore, targeted *Pistacia* supplementation during captive breeding is proposed as a promising hypothesis that requires further experimental validation to confirm its efficacy in reducing skeletal deformities. Furthermore, this diet-driven “microbiome stewardship” offers a potentially transferable conservation paradigm to mitigate captivity-induced physiological and skeletal disorders in other endangered avian species.

## Data Availability

The datasets generated during this study are available at the China National Center for Bioinformation (CNCB, https://www.cncb.ac.cn/) under BioProject PRJCA062213.
